# Monitoring Training and Recovery during a Period of Increased Intensity or Volume in Recreational Endurance Athletes

**DOI:** 10.3390/ijerph18052401

**Published:** 2021-03-01

**Authors:** Olli-Pekka Nuuttila, Ari Nummela, Keijo Häkkinen, Santtu Seipäjärvi, Heikki Kyröläinen

**Affiliations:** 1 Faculty of Sport and Health Sciences, University of Jyväskylä, 40014 Jyväskylä, Finland; keijo.hakkinen@jyu.fi (K.H.); heikki.kyrolainen@jyu.fi (H.K.); 2 KIHU—Research Institute for Olympic Sports, 40101 Jyväskylä, Finland; ari.nummela@kihu.fi; 3 Department of Psychology, Centre for Interdisciplinary Brain Research, University of Jyväskylä, 40014 Jyväskylä, Finland; santtu.m.seipajarvi@jyu.fi

**Keywords:** endurance performance, running, training load, heart rate variability

## Abstract

The purpose of the study was to examine the effects of progressively increased training intensity or volume on the nocturnal heart rate (HR) and heart rate variability (HRV), countermovement jump, perceived recovery, and heart rate-running speed index (HR-RS index). Another aim was to analyze how observed patterns during the training period in these monitoring variables were associated with the changes in endurance performance. Thirty recreationally trained participants performed a 10-week control period of regular training and a 10-week training period of either increased training intensity (INT, n = 13) or volume (VOL, n = 17). Changes in endurance performance were assessed by an incremental treadmill test. Both groups improved their maximal speed on the treadmill (INT 3.4 ± 3.2%, *p* < 0.001; VOL 2.1 ± 1.8%, *p* = 0.006). In the monitoring variables, only between-group difference (*p* = 0.013) was found in nocturnal HR, which decreased in INT (*p* = 0.016). In addition, perceived recovery decreased in VOL (*p* = 0.021) and tended to decrease in INT (*p* = 0.056). When all participants were divided into low-responders and responders in maximal running performance, the increase in the HR-RS index at the end of the training period was greater in responders (*p* = 0.005). In conclusion, current training periods of increased intensity or volume improved endurance performance to a similar extent. Countermovement jump and HRV remained unaffected, despite a slight decrease in perceived recovery. Long-term monitoring of the HR-RS index may help to predict positive adaptations, while interpretation of other recovery-related markers may need a more individualized approach.

## 1. Introduction

The rapid development of wearable technology has allowed for frequent monitoring of training and recovery. For example, heart rate measures during endurance training and rest are widely used, not only among elite and competitive athletes, but also in recreational athletes. While more measurement devices are available, it would be important to understand the practical relevance of the results, and how to interpret obtained results in the right context [1].

The main purpose of the monitoring process is to ensure that the body is adapting to the training stimulus and the training load is appropriate for the individual [2]. Another role of monitoring is to ensure sufficient recovery between training sessions and periods. Recovery and the training state can be analyzed from many perspectives, including assessments of performance [3], physiological markers such as hormone concentrations [4] or heart rate variability [5], and perceived estimations of the recovery [6]. Recovery-based training has been studied recently among multiple populations. Individually adjusted training-based on resting heart rate variability (HRV) has induced superior improvements in maximal endurance performance [7] and VO_2max_ [8] compared to pre-planned training in recreationally trained participants.

Responses to short-term [9] and long-term training periods [10] may vary quite a lot between individuals. Individual changes in endurance performance after a standardized endurance training program can range from slightly negative up to even a 20–30% improvement [9,11]. Multiple factors can explain the differences in the adaptation including, for example, genetics, training status, sleep, nutrition, and the recovery state [12]. It has been suggested that individualized training prescriptions may diminish variation in the adaptation [13].

It seems reasonable to assume that monitoring training and recovery could help athletes and coaches to react if an undesirable response would be detected. To the best of our knowledge, no studies have previously examined the recovery and training state in recreational athletes during an endurance training period of increased intensity or volume from a multidisciplinary point of view. Therefore, the purpose of the present study was first to examine the effects of increasing either intensity or volume on nocturnal heart rate and HRV, endurance and neuromuscular performance, and perceived recovery. Another aim was to analyze whether observed performance and recovery patterns during the training period could differentiate the low-responders from the responders. It was hypothesized that an increased volume of low-intensity training would impair neuromuscular performance assessed by countermovement jump [14] but increase the activity of the cardiac parasympathetic nervous system measured as HRV [15], unlike high-intensity training. It was also hypothesized that maintenance of stable recovery during the long-term training period as well as an improvement in the submaximal estimation of endurance performance may differentiate responders from low-responders.

## 2. Materials and Methods

### 2.1. Participants

A total of 42 recreationally endurance-trained 20–45 years old men (*n* = 21) and women (*n* = 21) were recruited for the study. There were five dropouts during the control period due to injuries (*n* = 3), illness (*n* = 1), and personal reasons (*n* = 1). During the training period, three dropouts occurred due to illness (*n* = 1) and personal reasons (*n* = 2). Four participants were excluded from the final analysis due to improper training adherence (<90% of the main sessions), leaving 30 participants in total for the final analysis. Participants were divided into the intensity-group (INT: 8 men, 5 women) and volume-group (VOL: 8 men, 9 women) at the end of the control period. The baseline characteristics of both groups are presented in Table 1. The study protocol was approved by the Ethical Committee of the University of Jyväskylä.

### 2.2. Study Design

The study consisted of two separate 10-week periods. During the first period subjects continued their typical training on their own (control period), while during the second period training was modified according to the group (training period). Laboratory tests, including incremental running tests on a treadmill and serum hormone analyses, were performed at the beginning of the control period (Ctrl), between the control and training periods (Pre), and at the end of the training period (Post). During the whole study period, the participants recorded weekly nocturnal heart rate (control period: 29 ± 3 nights, training period: 29 ± 4 nights), performed countermovement jump tests (control period: 8.9 ± 1.1 times, training period: 9.3 ± 1.0 times), collected training data from all endurance exercises (heart rate and speed), and filled a training log. Individual reference values for the recovery measurements and training characteristics (intensity and volume) were analyzed as an average of the control period. Weeks including illnesses were excluded from the analysis to avoid distorting the results. At the end of the control period, the participants were divided into two groups based on their background information (treadmill test performance, age, gender) and training characteristics. The INT-group increased the proportion of training sessions above the first lactate threshold, while the VOL-group increased the endurance training volume (low-intensity) during the training period.

### 2.3. Laboratory Tests

Fasting measurements: Fasting measurements were performed after 12 h of fasting and individually at the same time of the day (8:00–9:15 A.M.). Body mass and body fat percentage were measured with InBody770-analyser (Biospace Co. Ltd., Seoul, Korea). Blood samples were taken in a sitting position from the antecubital vein into 7 mL serum tubes using standard laboratory procedures. Whole blood was centrifuged at 2000 G rcf (Megafuge 1.0 R, Heraeus, Hanau, Germany) for 10 min, and after that serum was removed and frozen at −20 degrees until the final analysis. Serum cortisol was analyzed with chemical luminescence technique (Immulite 2000 XPi, Siemens, New York City, NY, USA). The sensitivity of cortisol assay was 5.5 nmol/L and the intra-assay coefficients of variation 3.6%. Free testosterone was analyzed with the ELISA-method (DYNEX DS 2 ELISA processing system, DYNEX Technologies, Chantilly, VA, USA). The sensitivity of free testosterone assay was 0.06 pmol/L and the intra-assay coefficient of variation was 3.6%.

Incremental treadmill test: An incremental treadmill test was performed on a treadmill (Telineyhtymä Oy, Kotka, Finland) always at the same time of the day (±2 h) within-participant. Starting speed was set to 7 or 8 km/h for women and 8 or 9 km/h for men. The starting speed was based on the background information of the participants to allow a reliable estimation of lactate thresholds and was kept similar in all tests. Three-minute stages were used, and speed increased by 1 km/h after every stage. Between the stages, the treadmill was stopped (15–20 s) for drawing the fingertip blood lactate samples. Inclination was kept constant at 0.5 degrees through the whole test. Oxygen consumption was measured breath by breath (OxygonPro, Jaeger, Hochberg, Germany) and heart rate was monitored with Garmin Forerunner 245M (Garmin Ltd., Schaffhausen, Switzerland). Maximal oxygen uptake (VO_2max_; mL/kg/min) was defined as the highest 60 s average of oxygen consumption. Due to technical issues regarding the gas analyser, reliable oxygen consumption values were available only from the control and pre-tests. Maximal running speed (vMax) of the test was defined as the highest completed speed, or if the stage was not finished, as a speed of the last completed stage (km/h) + (running time (s) of the unfinished stage − 30 s)/(180 − 30 s) × 1 km/h. The first lactate threshold (vLT1) and the second lactate threshold (vLT2) were determined based on blood lactate changes during the test [16]. The vLT1 was set at 0.3 mmol/L above the lowest lactate value and vLT2 at the intersection point between (1) a linear model between vLT1 and the next lactate point and (2) a linear model for the lactate points with the lactate increase of at least 0.8 mmol/L.

### 2.4. Training

Control period: A 10-week control period began after the control tests. During the control period, the participants were advised to continue their regular training in terms of volume and intensity. However, they were advised to be at the recovered state at the end of the control period.

**Figure 1:**
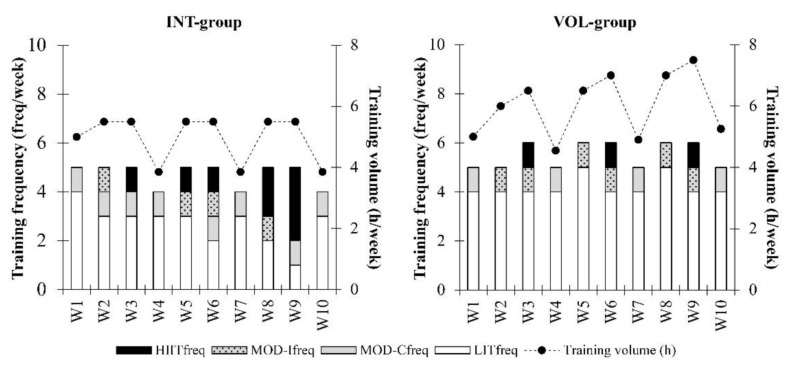
Training program progression of a representative participant in the INT- and VOL-groups. INT, Intensity-group; VOL, Volume-group; HIITfreq, frequency of high-intensity interval training; MOD-I, frequency of moderate-intensity interval training; MOD-Cfreq, frequency of moderate-intensity continuous training; LITfreq, frequency of low-intensity training.

Training period: During the 10-week training period, the participants of the INT- and VOL-groups utilized individually scaled training programs. The aim was to increase progressively training load by either increasing the proportion of moderate and high-intensity sessions or volume of the training. After one easier week, during which the participants were familiarized with the predefined training, it was periodized into three three-week mesocycles of two intensive weeks followed by one recovery week (70% volume of the preceding week, only one moderate-intensity-session). The goal of the INT-group was to progressively increase the proportion of training above the first lactate threshold compared to the average of the control period, while maintaining the total endurance training volume the same. Progression started from one additional session and led to three additional sessions during the intensive training weeks, accounting for 10 sessions in total. Furthermore, the intensity of these sessions progressed from moderate-intensity training towards high-intensity training. The goal of the VOL-group was to progressively increase the volume of low-intensity training compared to the control period from 20% to 50% during intensive weeks while maintaining the volume of moderate and high-intensity training the same. Volume was increased primarily by adding duration to each training session, and weekly training frequency was kept similar. The training progression during the training period is illustrated in Figure 1.

The training program included low-intensity training (LIT) below the first lactate threshold, moderate-intensity training (MOD) between the first and second lactate threshold, and high-intensity training (HIT) above the second lactate threshold. Session intensity was controlled by the heart rate. The duration of the training sessions was individually determined in accordance with typical sessions performed during the control period. LIT-sessions consisted of basic sessions (30–75 min) and long sessions (>75 min). MOD-sessions consisted of long intervals (2–4 × 10–15 min) or continuous running (20–60 min). HIT-sessions consisted of 3–6 min intervals with 2:1 work: relief-ratio, and 15–30 min accumulated time in the high-intensity during the session. Interval sessions always included low-intensity warm-up and cool-down. The training was performed mainly by running. To avoid the risk of overuse injuries, alternative training modes (cycling, roller-skiing, swimming) were allowed with volumes similar to the control period. Subjects were advised not to change the amount or content of their typical strength training during the study period (control period: 0.3 ± 0.3 sessions/week, training period: 0.2 ± 0.3 sessions/week).

### 2.5. Training and Recovery Monitoring

Training data: The participants used the Garmin Forerunner 245M heart rate monitor (Garmin Ltd., Schaffhausen, Switzerland) during each endurance training session. Measured training data was regularly sent to the research group for further analysis. Distance covered (km) and time spent at each training intensity (LIT: HR < LT1, MOD: HR = LT1-LT2, HIT: HR > LT2) were analyzed from the sessions. Additionally, the heart rate-running speed index (HR-RS index) [17] was analyzed from all continuous-type running exercises. Sessions that were ran on trails or in the forest were excluded from the analysis. HR-RS index was calculated based on the session average running speed (S_avg_) and heart rate (HR_avg_) with the following equation:

HR-RS index = S_avg_ − (HR_avg_ − HR_standing_)/k

k = (HR_max_ − HR_standing_)/S_peak_

HR_standing_ was estimated by adding 26 bpm to the resting HR (average nocturnal HR during the control period) similar to Vesterinen et al. [17]. S_peak_ and HR_max_ were determined based on the first incremental treadmill test.

Training log: The participants wrote down the basic characteristics of each session, including training mode, session goal, session duration, distance covered, and optional own comments on a training log. In addition, session RPE [18] and recovery state during the training session [6] were estimated from each training day on a 0–10 scale.

Heart rate and heart rate variability: Nocturnal heart rate and HRV were measured three nights per week (2 weekdays and 1 weekend) with Firstbeat Bodyguard 2 device (Firstbeat Technologies Ltd., Jyväskylä, Finland). The participants were advised to start the measurement when going to sleep and stop the measurement right after awakening. The data was analyzed using Firstbeat Analysis Server software (version 7.5). The HRV analysis was performed by calculating the second-by-second HRV indices using the short-time Fourier transform method. Average heart rate and the natural logarithm of high-frequency power (lnHF ms^2^, 0.15–0.40 Hz) were obtained from the standardized time period of 0:30–4:30 after going to bed, similar to previous studies using nocturnal heart rate recordings [16,19]. lnHF was chosen as a representative HRV parameter, because it can be used to monitor changes in cardiac vagal control [20], it has been used in endurance training guidance [21], and it has also been associated with endurance training adaptation [22].

Countermovement jump: The countermovement jump (CMJ) test was performed once per week at home conditions. In the test, the participants performed three maximal attempts with a 1-min recovery. They were advised to perform the test after a short standardized warm-up at the same time of the day (±1 h), and on the same day of the week, before any physical activity. The jumps were videotaped with the mobile phone, which should have at least 120 frames per second video feature. Participants were instructed to use a camera angle from the front (about 1.5 m from the jumper) that would allow strict estimation of the first frame in which no foot touches the ground, and first frame had at least one foot contact again. Videos were sent to the research group and jumps were analyzed by the same person with a validated MyJump2-application [22]. Average jumping height (cm) of two best jumps were used in the data analysis.

### 2.6. Statistical Analysis

All values are expressed as mean and standard deviation (SD). The normal distribution of the data was verified with the Shapiro-Wilk test. In the laboratory measurements, differences between time points (control, pre, post) and groups were analyzed by a repeated measures ANOVA. In the case of a significant main effect or interaction, a Bonferroni post hoc test was used. For the monitoring variables, within-group comparisons between the control and training periods were assessed by paired samples *t*-test with absolute values and between-group comparisons by independent samples t-test with relative changes. Training characteristics (absolute and relative training intensity distribution) were not normally distributed, thus the Wilcoxon signed-rank -test was used for comparisons between the control period and training period. To further analyze changes in the monitoring variables that did not differ between groups (lnHF, CMJ, HR-RSi, perceived recovery), participants were retrospectively divided into two groups based on the relative change in the maximal treadmill performance (vMax). vMax was chosen to present endurance training adaptation, as it is closely related to maximal endurance performance in a wide range, and it has also been used in a previous study [16]. The low-responder group (n = 7, range −1.8 to 0.0%) included participants with no change or decrease in performance, while the responder group (n = 7, range +4.1 to +11.3%) included participants with a greater improvement than mean response after the control period + 1 × SD (>4.0%). Group comparisons were performed with the Mann-Whitney U-test and Bonferroni adjustments. The smallest worthwhile change (SWC) was calculated by multiplying the within-participant CV of each monitoring variable during the control period by 0.5 [23], except for the HR-RS index, where between-participant SD during the control period was multiplied by 0.5. The same average values were used for all participants. To examine the magnitude of observed changes, the effect size (ES) of within-group absolute differences and between-group differences in the relative changes was calculated as Cohen’s d for the main variables. The magnitude of changes was stated as <0.2 trivial, 0.2–0.5 small, 0.5–0.8 moderate, and >0.8 large. After nonparametric tests, effect size was calculated with a formula: ES = Z/√n, where Z is the z-score, and n are the number of observations. The significance level was set to *p* < 0.05. The analysis was performed with Microsoft Excel 2010 (Microsoft Corporation, WA, USA) and IBM SPSS Statistics v.26-programs (SPSS Inc, Chicago, IL, USA).

## 3. Results

### 3.1. Training

No differences were observed between the groups in the training characteristics of the control period. Average weekly training characteristics are presented in Table 2.

### 3.2. Laboratory Measurements

A significant main effect of time (*p* < 0.001) was found in vMax, vLT1, and vLT2. No differences were observed between the control and pre-tests in any of the laboratory measurements in neither of the groups. vMax improved in both groups after the training period (INT 3.4 ± 3.2%, *p* < 0.001, ES = 0.37; VOL 2.1 ± 1.8%, *p* = 0.006, ES = 0.18). In addition, running speed at the first lactate threshold (INT 4.6 ± 6.1%, *p* = 0.006, ES = 0.34; VOL 8.4 ± 5.5%, *p* < 0.001, ES = 0.62) and the second lactate threshold (INT 3.0 ± 3.1%, *p* = 0.007, ES = 0.29; VOL 3.7 ± 3.6%, *p* < 0.001, ES = 0.27) increased in both groups. In serum hormone concentrations, no significant main effect or interaction was observed. The absolute results of endurance performance and serum hormone concentrations are presented in Table 3.

### 3.3. Training and Recovery Monitoring

**Figure 2:**
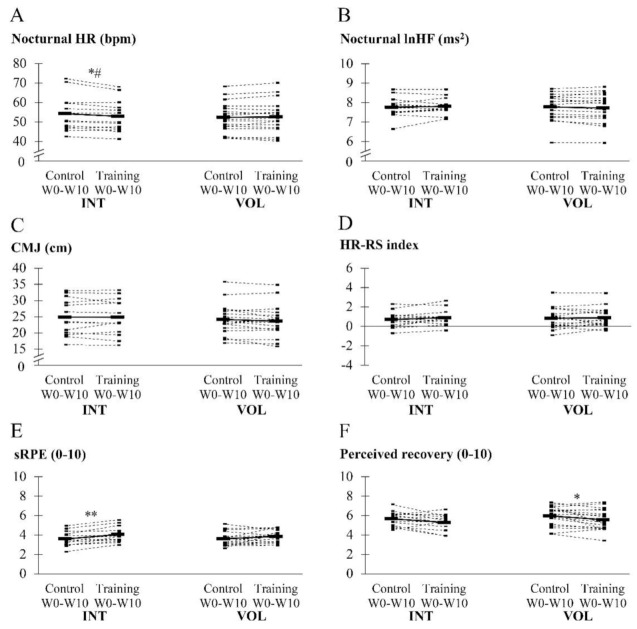
Individual average values during the control and training period in the nocturnal heart rate (HR) and heart rate variability (lnHF), countermovement jump (CMJ), heart rate-running speed index (HR-RS index), session RPE (sRPE) and perceived recovery. * *p* < 0.05 in within-group comparison to control, ** *p* < 0.01 in within-group comparison to control. # = *p* < 0.05 in between-group comparison with relative values.

Individual averaged values of the monitoring variables are presented in Figure 2. Significant differences between the control and training periods were observed in the session RPE of INT (*p* = 0.001, ES = 0.58), perceived recovery of VOL (−6.3 ± 10.1%, *p* = 0.021, ES = −0.43) and nocturnal heart rate of INT (*p* = 0.016, ES = −0.14). The relative change of nocturnal heart rate was significantly different between the groups (INT −2.1 ± 2.6% vs. VOL 0.4 ± 2.5%, *p* = 0.013, ES = −0.99). In addition, perceived recovery tended to decrease in INT (−6.1 ± 11.4%), *p* = 0.056, ES = −0.45). Small to moderate effect sizes were observed when relative changes were compared between the groups in CMJ (INT 0.0 ± 5.0% vs. VOL −2.3 ± 5.1%, ES = 0.46), lnHF (INT 0.7 ± 2.9% vs. −0.6 ± 1.8%, ES = 0.54), session RPE (INT 13.9 ± 12.4% vs. VOL 8.8 ± 18.4%, ES = 0.32), and in absolute changes of the HR-RS index (INT 0.2 ± 0.4 vs. 0.0 ± 0.5, ES = 0.34).

### 3.4. Comparison between Responders and Low-Responders

**Figure 3:**
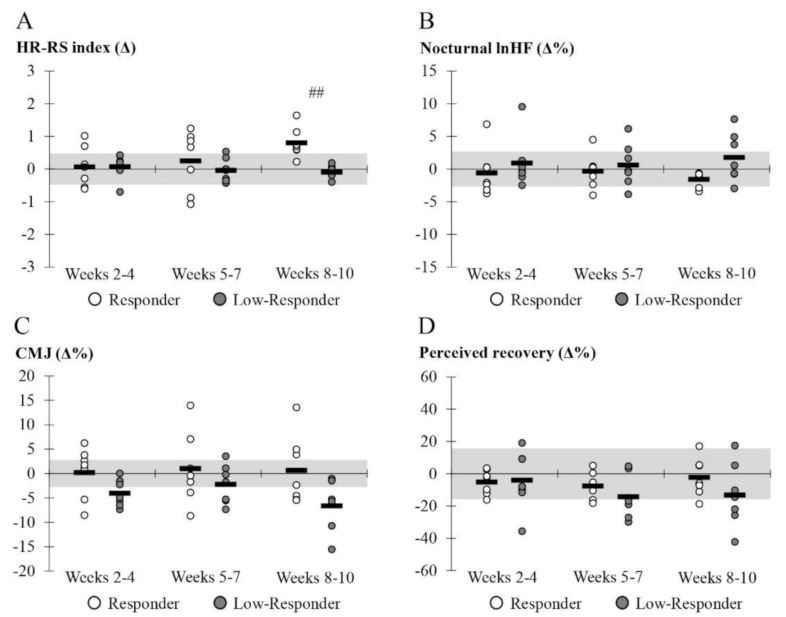
Mean (black line) and individual values (dots) in the relative changes compared to the control period in the nocturnal HRV (lnHF), heart rate-running speed index (HR-RS index), countermovement jump (CMJ), and perceived recovery. The gray area represents the smallest worthwhile change. ## *p* < 0.01 in between-group comparison.

For further analysis, the both groups were combined so that the participants were retrospectively divided into the subgroups of low-responders and responders. When the subgroups were compared across the training period, the only significant difference was observed in the HR-RS index during the last mesocycle (*p* = 0.005, ES = −0.84). In weeks 8–10, small to moderate between group effect sizes were also observed in lnHF (ES = −0.56), perceived recovery (ES = −0.32), and CMJ (ES = −0.50). Individual values in the relative changes across the training period are presented in Figure 3.

## 4. Discussion

The main findings of the study were that the present 10-week endurance training period of either increased intensity or volume improved endurance performance quite similarly, and all participants improved lactate threshold and/or maximal running speed in the incremental treadmill test. The monitoring variables were affected rather marginally at the group level, but there was a lot of variations between individuals in the observed responses during the training period, regardless of the type of training performed. An increasing trend in the HR-RS index seems to be desirable when monitoring endurance training, while the interpretation of other recovery-related parameters, what kind of change should be regarded as worthwhile, as well as the choice of the monitoring variables may need a more individualized approach.

The training protocols were planned so that the training load would progressively increase either via intensity or volume, and training would be the most demanding at the end of the training period. The VOL-group increased their training volume approximately by 20%, while the INT-group increased the proportion of HIT-training from 2 to 7%. It is fair to assume that the training load was somewhat appropriate for the participants, as all individuals improved their maximal performance or running speed at the lactate thresholds, and none of the participants could be regarded as overreached at the time of post-tests. This was also supported by the unchanged concentrations of serum cortisol or free testosterone. Both groups improved performance almost identically, although a moderate between-group effect was observed in the improvement of the first lactate threshold in the favor of the VOL-group, and in turn, a similar between-group effect favoring the INT-group was observed in the improvement of maximal treadmill performance. The observed changes were mainly in line with previous studies using a similar type of training approach [16,24]. Regarding the training intensity distribution, typically 80% LIT and 20% MOD/HIT are stated to be a recommendable basis for endurance athletes [25]. In the present study, both group’s average value was quite close to that. However, certain types of training distribution, such as high training volume in the INT-group, or high amount of moderate and high-intensity sessions in the VOL-group, may have been unfavorable during the training period, because the training was individually scaled based on control period characteristics.

Different types of heart rate measures are widely used in endurance training monitoring [26], and resting HRV is particularly suggested to be a useful marker when assessing recovery [5]. While acute responses in HRV are mostly related to training intensity, and sessions above the first lactate threshold delays parasympathetic reactivation compared to low-intensity sessions below the first lactate threshold [27,28], long-term responses to different training strategies seem to be more complicated. In the present study, no systematic changes during the training period were observed in HRV neither in INT nor in VOL. At an individual level, both decreasing and increasing responses were observed, thus illustrating the individuality of HRV. It is important to notice that both an increase and a decrease in HRV may be a sign of fatigue and overreaching [29,30], which is why values outside the SWC-range in both directions could be a negative sign when monitoring recovery [13]. Plews et al. [15] have previously suggested that high-volume low-intensity training induces increases in HRV and positive changes in the balance of the autonomic nervous system. It is possible that in the present study, such findings were not found in VOL because the amount of the MOD- and HIT-training was quite high in some individuals (2–3 weekly sessions > first lactate threshold), and the total endurance training volume was much lower than in elite rowers training almost 20 h/week during high volume periods in the study by Plews et al. [15]. It is also important to note that HRV has mainly been studied during the morning measurements [15,29,30], which may induce different results compared to the nocturnal measurements. What can be said in favor of sleep time recordings is that they are not affected by external factors to the similar extent as awake recordings, thus theoretically allowing the most standardized period for the measurement [26]. In addition, sleep itself is a very important aspect of recovery [31] and therefore, HRV monitoring during sleep may provide additional information about the recovery process itself. While nocturnal recordings may have been challenging to implement frequently [26], wearable technology will most likely keep evolving, allowing more methods for feasible and valid assessments of HRV. It is probable that recreational athletes would especially prefer monitoring tools that would not require any extra effort or time.

Besides different recording times, multiple different variables could also be obtained from the heart rate measurements. A simple nocturnal heart rate reflects somewhat similar aspects of recovery as HRV [26]. Consequently, the heart rate has been affected acutely most by the intensity of the training [27], and after high-intensity interval exercise performed in the evening, responses in nocturnal heart rate may be even more severe than in HRV [32]. On the other hand, after long-term high-intensity training, heart rate may decrease significantly [19]. In the present study, the nocturnal heart rate slightly decreased in INT, and a significant difference between groups was also observed in the relative change from the control to the training period. Based on these and previous findings, the nocturnal heart rate may react more uniformly and sensitively to high-intensity training, both acutely [32] and in the long-term [19], compared to HRV when using nocturnal recordings. Whether this is associated with physiological factors such as changes in plasma volume or cardiac morphology [26] has yet to be studied.

Submaximal exercise tests are another typical way to estimate the training state [3] and adaptations [33] to endurance training. Maximal performance is very difficult to assess regularly without disturbing the normal training process, and therefore, endurance athletes need to settle for indirect and submaximal estimations of maximal performance, typically based on the relation between heart rate and running speed [17]. In the present study, the HR-RS index that was calculated from all continuous types of training sessions was used as an indirect estimation of endurance performance. Despite the improved maximal performance, no significant difference was observed between the control and training period in either of the groups. Previously, increments in the HR-RS index [17] and running speeds of the submaximal running tests [33] have both correlated with the change in maximal running performance. The lack of significant change in the present study may relate to the long averaging period (10 weeks) of the results. Since there possibly are some fluctuations in the HR-RS index due to changes in training load, sessions that are performed at the recovered state may predict changes in performance more accurately. One clear limitation in submaximal tests relying on heart rate is that similar to resting HRV, the same type of responses (decrease in submaximal heart rate) could be found after positive training adaptation [33] and during functional overreaching [29]. Another challenge in the HR-RS index is that environmental factors such as the amount of ascent during the session or outdoor temperature may both affect the relation between the heart rate and running speed. In the current study, only continuous sessions were used in the analysis similar to Vesterinen et al. [17]. As increased intensity improves the accuracy of indirect estimations of maximal endurance performance [33], methods that would allow estimations from high-intensity interval training could also be advantageous.

Neuromuscular characteristics play an essential role in distance running performance [34,35]. Especially in running, which induces high stress in the musculoskeletal tissues of the lower limbs, mechanical fatigue caused by training may also relate to overuse injuries [36]. It would therefore seem logical that maintaining or even improving neuromuscular performance would be of importance to endurance athletes. In the present study, the CMJ performance was monitored as an indicator of neuromuscular recovery once a week, similar to Balsabore–Fernandez et al. [14], who found that increased training load and running volume were associated with impaired CMJ during a 39-week follow-up study. Furthermore, the authors found that better CMJ was accompanied by better performance in running competitions. Bachero–Mena et al. [37] also found that during the competitive season, positive trends in both CMJ and running performance were observed in middle-distance runners. In the present study, no significant differences were found between or within the groups. However, based on the effect size of the observed changes, it seems that an increase in endurance training volume may have a slightly higher risk to impair neuromuscular performance than the increase in intensity of endurance training. The training of middle-distance runners [37] and high-level athletes [14] is likely more demanding for the neuromuscular system, and responses to training could, therefore, be more distinct compared to the population of the present study. It should also be evaluated in more detail whether there are more sensitive markers to monitor neuromuscular aspects of recovery in recreational runners, such as sprint tests or variables obtained from half-squat, which have reacted to changing training load in elite runners [38].

In addition to performance and physiological markers, recovery and training state could also be assessed from a subjective perspective. In the systematic review of Saw et al. [39], subjective markers were suggested to be more sensitive than objective measures to acute and chronic changes in the training load. Haaf et al. [40] have even argued that subjective markers could predict the overreaching state after a few days of intensive cycling event. In the current study, perceived recovery was monitored with a simple 0–10 scale [6]. Perceived recovery slightly decreased during the training period in VOL and tended to decrease in the INT, suggesting that an increased training load had at least a minor effect on the subjective feeling of recovery. Also, average session RPE increased in the INT, while in the VOL, it remained the same. Although the increase in RPE may relate to exercise-induced cumulative fatigue [41], in the present study the difference was probably mainly the outcome of the increased amount of high-intensity sessions. When comparing the results to previous studies, differences in the questionnaires that have been utilized may also explain the results. Although more comprehensive surveys could provide additional and more precise information about the recovery status of an athlete, a simple 0–10 scale [6] was used to allow monitoring of perceived recovery on a daily basis and with the setting that would be practical and realistic to utilise in long-term.

When the low-responders and responders were compared, none of the used monitoring variables were able to predict positive adaptation unequivocally, and especially at the beginning of the training period, no significant differences between the subgroups were found. However, at the end of the training period, an increase in the HR-RS index seemed to differentiate positive responders and low-responders in maximal running performance. Although no other marker exclusively differentiated low-responders from responders, several trends could most likely be stated as being unfavorable. Increased nocturnal HRV compared to the smallest worthwhile change (3 vs. 0 individuals), as well as decreased perceived recovery (4 vs. 1 individuals) and neuromuscular performance (5 vs. 3 individuals) were all more frequent observations among the low-responders than responders during the last mesocycle of the training period. The results of the current study most likely illustrate how the sensitivity of different monitoring variables response to variation in training load or fatigue may vary among individuals. Furthermore, interpretation of the results—what kind of change should be regarded as worthwhile—as well as the choice of the monitoring variables that may need to be evaluated individually [42]. One unsolved and somewhat critical question regarding the interpretation is how often individual reference values should be updated. Another important aspect is to ensure the quality of the data as well as the adequate frequency of the assessments of each variable. Rather than relying on one marker only, a multifaceted approach may help to contextualize observed patterns [30] improving the quality of the monitoring process.

### Study Limitations

The study population consisted of recreationally trained endurance athletes with slightly varying training background and age. Further studies are needed to study the usefulness of similar monitoring variables in more specific populations (e.g., untrained and elite-level athletes) and with larger sample sizes. In the low-responder vs. responder comparison, both groups were combined because the small sample size did not allow meaningful separate analysis. However, no significant differences were found between the groups in the changes of monitoring variables or training adaptation so the current division most likely did not affect the outcome. The study was performed under field conditions so that the participants trained and performed recovery measures by themselves, not in the laboratory. The circumstances were different compared to the strict laboratory conditions. However, the present setting most likely represents the usefulness of the chosen monitoring variables well in practice.

## 5. Conclusions

In conclusion, current training periods of increased intensity or volume improved endurance performance to the similar extent, and nocturnal HR and perceived recovery were the only monitoring variables that were affected by the training, while no changes at a group level were observed in HRV or CMJ performance. Based on comparison between responders and low-responders, continuous monitoring of training-related parameters, such as the HR-RS index, may help to predict whether an individual is adapting to training. The sensitivity of the recovery-related variables may vary between individuals, and interpretations, as well as choice of appropriate markers, may therefore need a more individualized approach.

## Figures and Tables

**Table 1:** Baseline characteristics of the participants at the beginning of the control period.

	INT (*n* = 13)	VOL (*n* = 17)
Age (yrs.)	38 ± 4	36 ± 6
Height (cm)	173 ± 11	172 ± 11
Body mass (kg)	72.0 ± 11.9	69.5 ± 11.8
Body fat (%)	17.4 ± 6.9	19.4 ± 7.7
VO_2max_ (mL/kg/min)	47.1 ± 5.6	47.2 ± 5.4
Training history (yrs.)	11 ± 10	10 ± 7

Values are presented as means ± SD. INT, intensity-group; VOL, volume-group; VO_2max_, maximal oxygen uptake.

**Table 2:** Average weekly training characteristics during the control and experimental training periods.

	INT (*n* = 13)		VOL (*n* = 17)	
	Control	Training	Control	Training
Training volume (h)	4.9 ± 1.7	4.8 ± 1.7	4.9 ± 1.4	5.7 ± 1.8 ***
Training frequency/week	4.6 ± 1.3	5.1 ± 1.7	4.6 ± 1.1	4.8 ± 1.1
Running volume (km)	31 ± 11	38 ± 16 *	34 ± 13	44 ± 14 ***
LIT (%)	77 ± 17	71 ± 12	75 ± 15	77 ± 12
MOD (%)	21 ± 16	22 ± 9	22 ± 13	19 ± 9
HIT (%)	2 ± 3	7 ± 6 **	3 ± 3	4 ± 3

INT, intensity-group; VOL, volume-group; LIT, low-intensity training below the first lactate threshold; MOD, moderate-intensity training between the first and the second lactate thresholds; HIT, high-intensity training above the second lactate threshold. *** *p* < 0.001, ** *p* < 0.01, * *p* < 0.05 different compared to the control.

**Table 3:** Laboratory test results at the beginning (Pre) and the end (Post) of the training period.

	INT (*n* = 13)		VOL (*n* = 17)		Effect Size
	Pre	Post	Pre	Post	INT vs. VOL (Δ% Pre-Post)
Endurance Performance					
vLT1 (km/h)	10.2 ± 1.3	10.7 ± 1.2 **	10.1 ± 1.3	10.9 ± 1.1 ***	−0.65 (moderate)
vLT2 (km/h)	12.7 ± 1.5	13.1 ± 1.4 **	12.5 ± 1.6	13.0 ± 1.5 ***	−0.19 (trivial)
vMax (km/h)	15.7 ± 1.4	16.2 ± 1.4 ***	15.5 ± 1.7	15.8 ± 1.8 **	0.50 (moderate)
Serum hormone concentrations					
Cor M (nmol/L)	343 ± 97	356 ± 90	363 ± 85	346 ± 110	0.27 (small)
fTesto M (pmol/L)	40 ± 25	36 ± 22	30 ± 21	28 ± 22	−0.03 (trivial)
fTesto:Cor	0.11 ± 0.06	0.10 ± 0.05	0.10 ± 0.05	0.09 ± 0.08	−0.39 (small)

Values are presented as means ± SD. vLT1, the speed at the first lactate threshold; vLT2, the speed at the second lactate threshold; vMax, maximal speed of the incremental treadmill test; Cor, serum cortisol; fTesto, serum free testosterone; fTesto:Cor, the ratio between serum free testosterone and cortisol; INT, Intensity-group; VOL, Volume-group. *** *p* < 0.001, ** *p* < 0.01, different compared to the pre, ES = Effect size as Cohen’s D.

## Data Availability

Data are available on request to the corresponding author according to the ethics approval of the local ethics committee and conditions of contract agreed with the funder.
